# Supplementary material for: Deciphering bat influenza H18N11 infection dynamics in male Jamaican fruit bats on a single-cell level
Source: Nat Commun. 2024 May 27;15:4500. doi: 10.1038/s41467-024-48934-6 (PMC11130286; doi:10.1038/s41467-024-48934-6)
Supplement: Supplementary file 1 — Supplementary Information [file 41467_2024_48934_MOESM1_ESM.pdf]

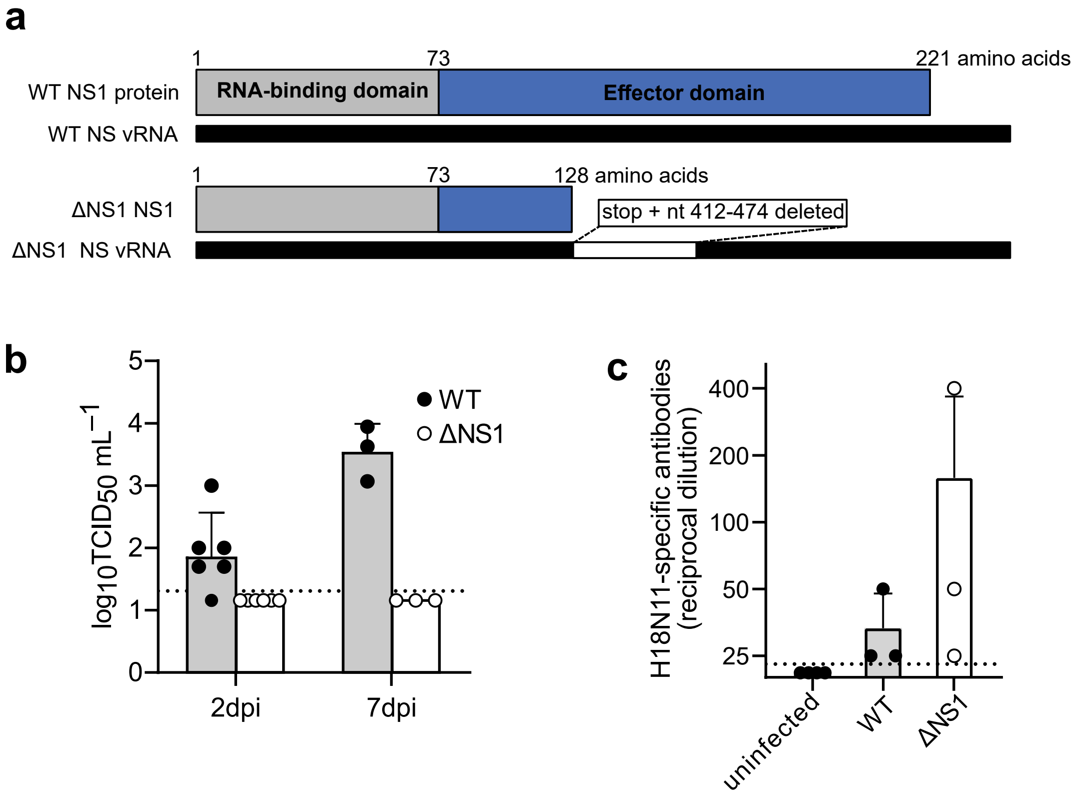

Supplementary Fig. 1: Virus shedding and antibody titers in infected Jamaican fruit bats. **a**, Cartoon showing the WT and truncated IAV NS1 segment as vRNA sequence and the resulting protein. **b**, Infectious virus in rectal swabs from WT (*n*= 6) and ΔNS1-infected bats (*n*= 6) were determined at the indicated time points. Data are the mean ± SD. Dashed line indicates detection limit. **c**, H18N11-specific antibodies from uninfected controls (*n*= 4), WT (*n*= 3) and ΔNS1-infected bats (*n*= 3) at 9 dpi were determined by an ELISA. Dashed line indicates detection limit. Source data are provided as a Source Data file.

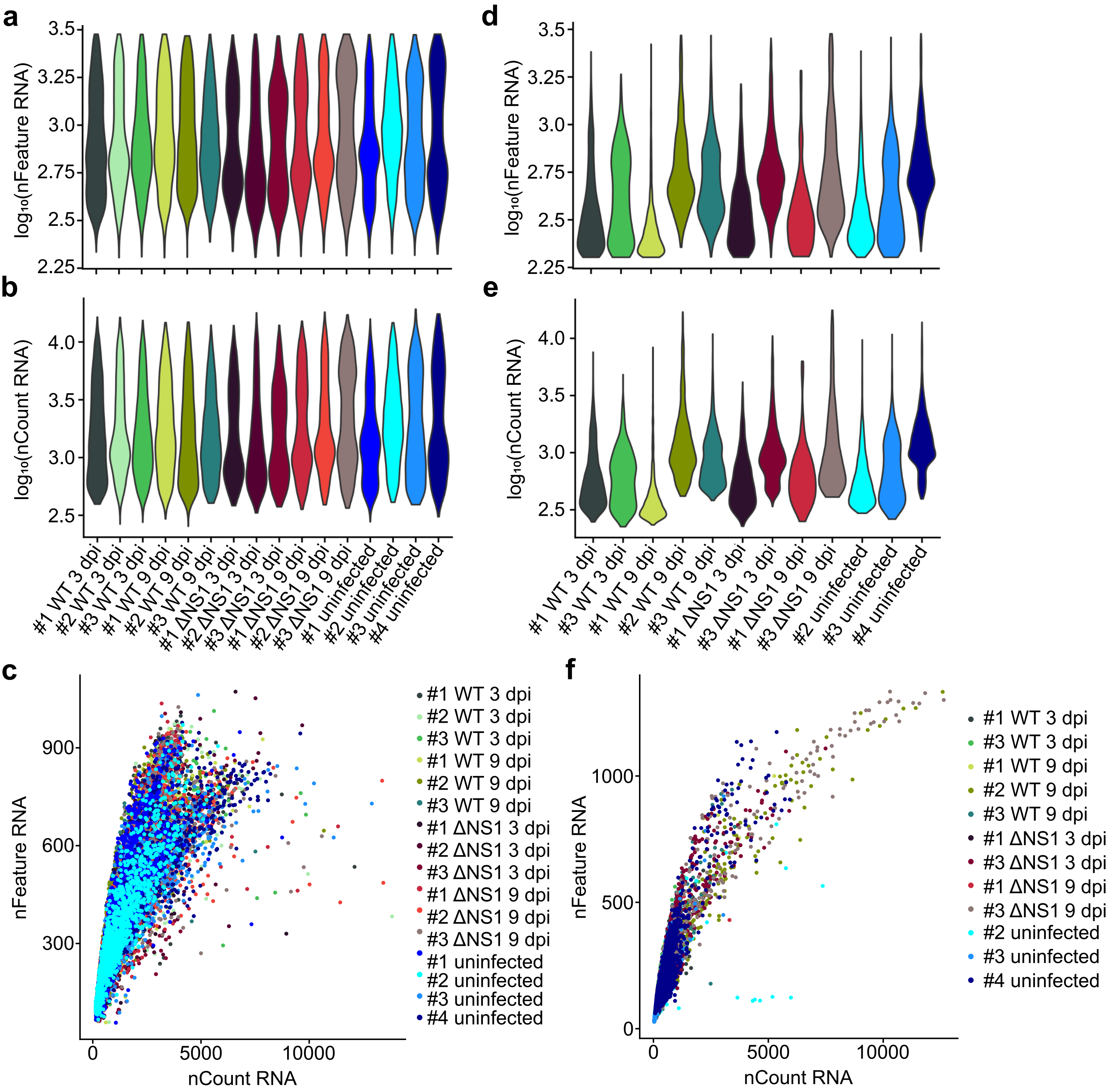

Supplementary Fig. 2: Quality control plots of the intestinal and mesenteric bat samples. a,**d**, Violin plot showing the overall distribution of the number of quantified genes per cell (nFeature) in the intestine (**a**) and mesentery (**d**) for each sample. **b**,**e**, Number of reads (nCount) of individual intestinal (**b**) and mesentery (**e**) samples (see method for the details regarding quality filters). **c**,**f**, Scatter plot showing the correlation between the nFeature and nCount from the top 5,000 most variable features for each sample in the intestine (c) and mesentery (f) (color coded). Source data are provided as a Source Data file.

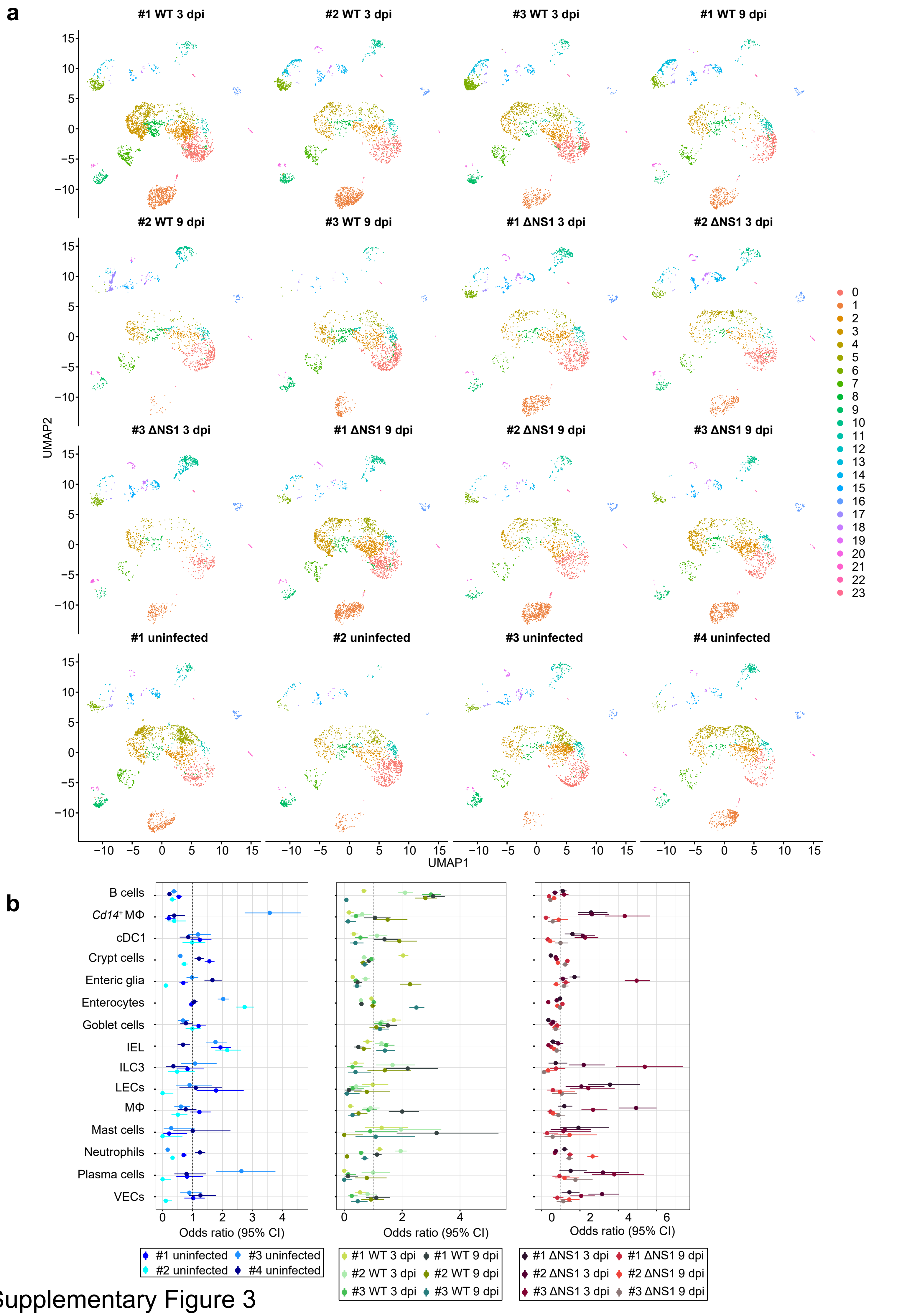

Supplementary Figure 3

Supplementary Fig. 3: Individual UMAPs and sample-wise changes in the cellular composition of the intestine. a, UMAP presentation of cells from the intestine of uninfected control bats or bats from the WT 3 dpi, WT 9 dpi, ΔNS1 3 dpi or ΔNS1 9 dpi infection group. Numbers indicate the Louvain clustering ID (color coded). **b**, Forest plot showing sample-wise enrichment of the indicated cell types in each bat as the odds ratio determined by the Fisher's exact test with a 'greater' alternative (one-sided). Error bars indicate 95% confidence interval. Source data are provided as a Source Data file.

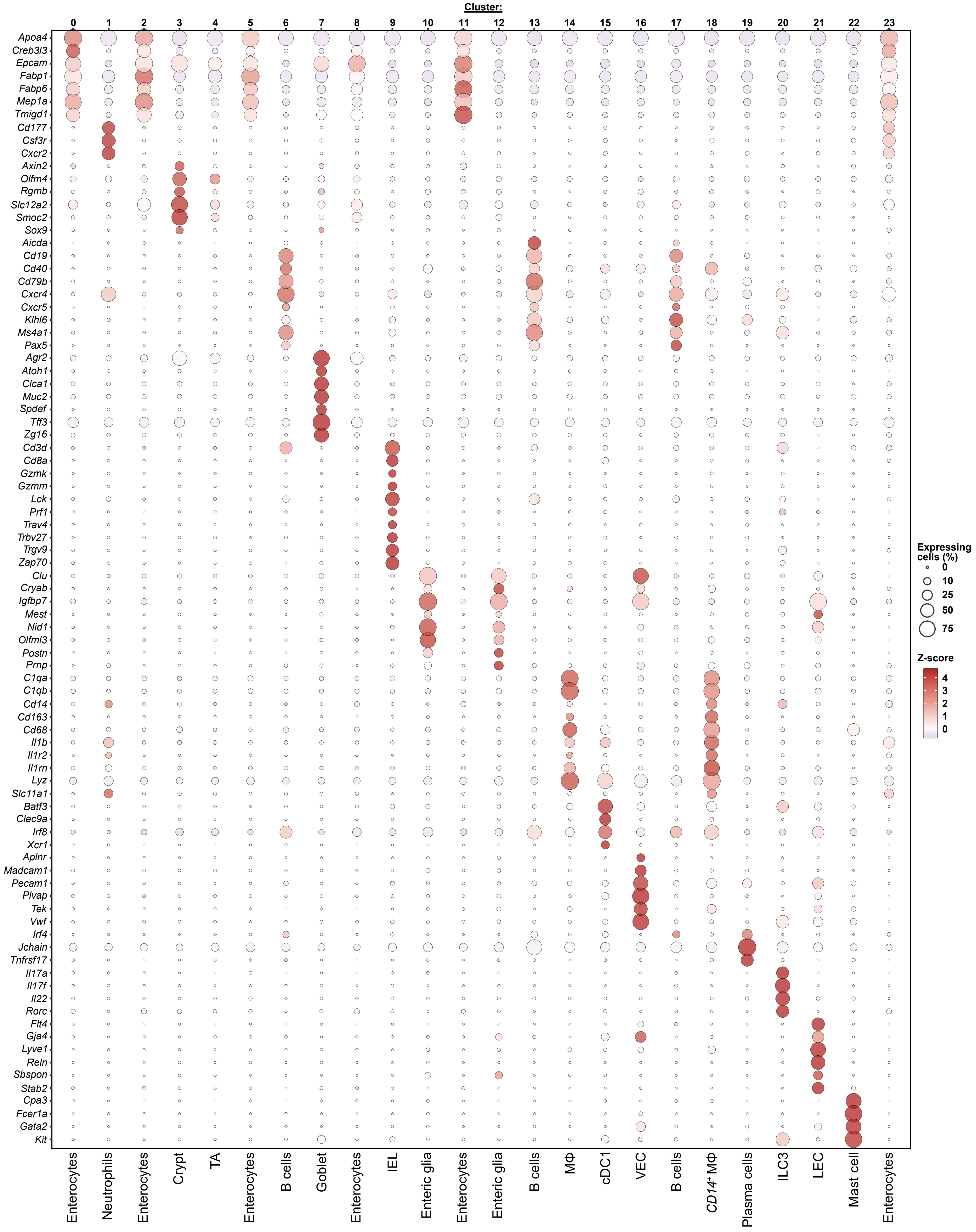

Supplementary Figure 4

Supplementary Fig. 4: Intestinal cell types defined in the study. Dot plot showing the expression of marker genes of intestinal cell types in the scRNA-seq dataset. Color scale shows the normalized log_2 expression. Source data are provided as a Source Data file.

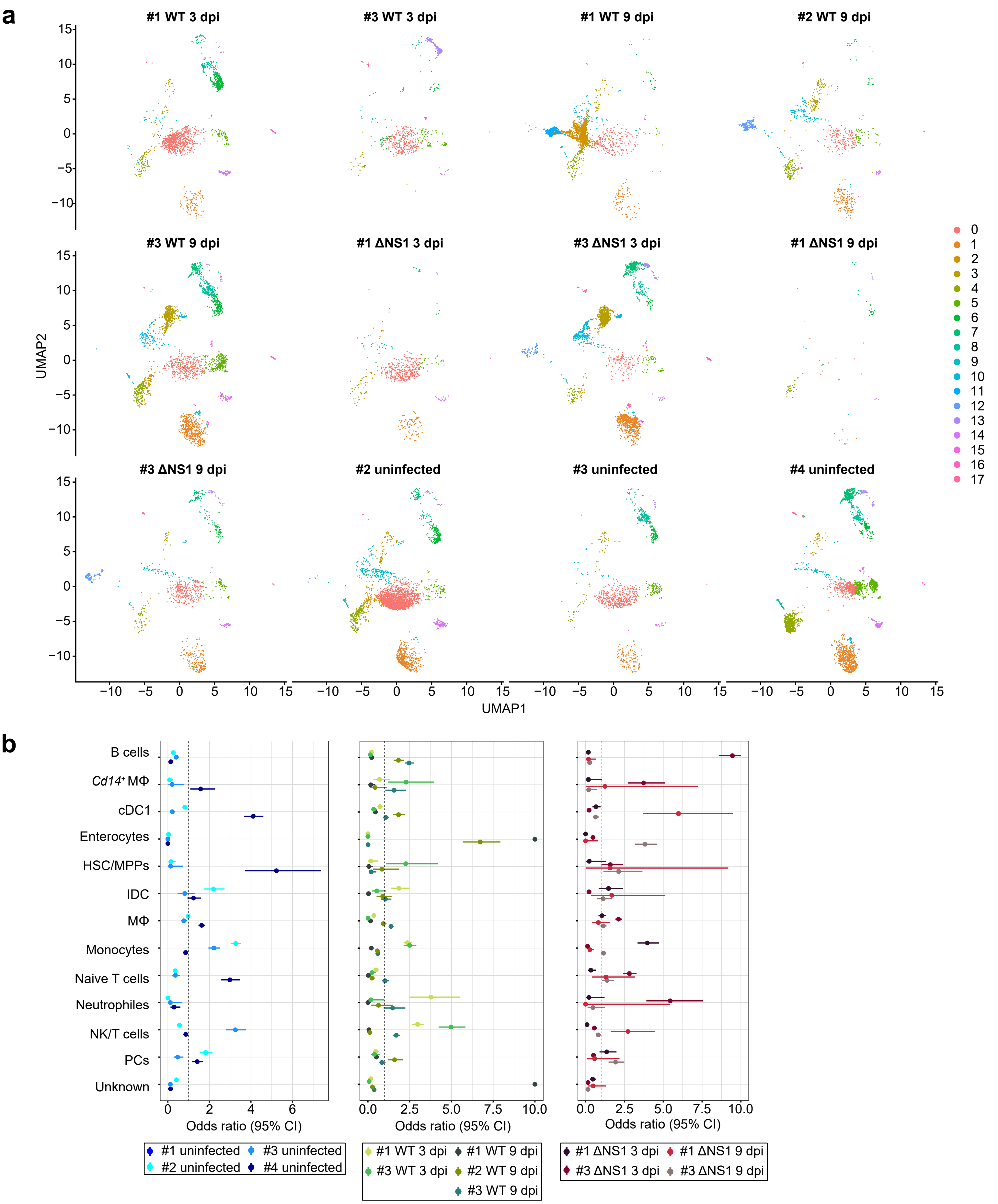

Supplementary Figure 5

Supplementary Fig. 5: Individual UMAPs and sample-wise changes in the cellular composition of the mesentery. **a**, UMAP presentation of cells from the mesentery of uninfected control bats or bats from the WT 3 dpi, WT 9 dpi, ΔNS1 3 dpi or ΔNS1 9 dpi infection group. Numbers indicate the Louvain clustering ID (color coded). **b**, Forest plot showing sample-wise enrichment of the indicated cell types in each bat as the odds ratio determined by the Fisher’s exact test with a 'greater' alternative (one-sided). Error bars indicate 95% confidence interval. Source data are provided as a Source Data file.

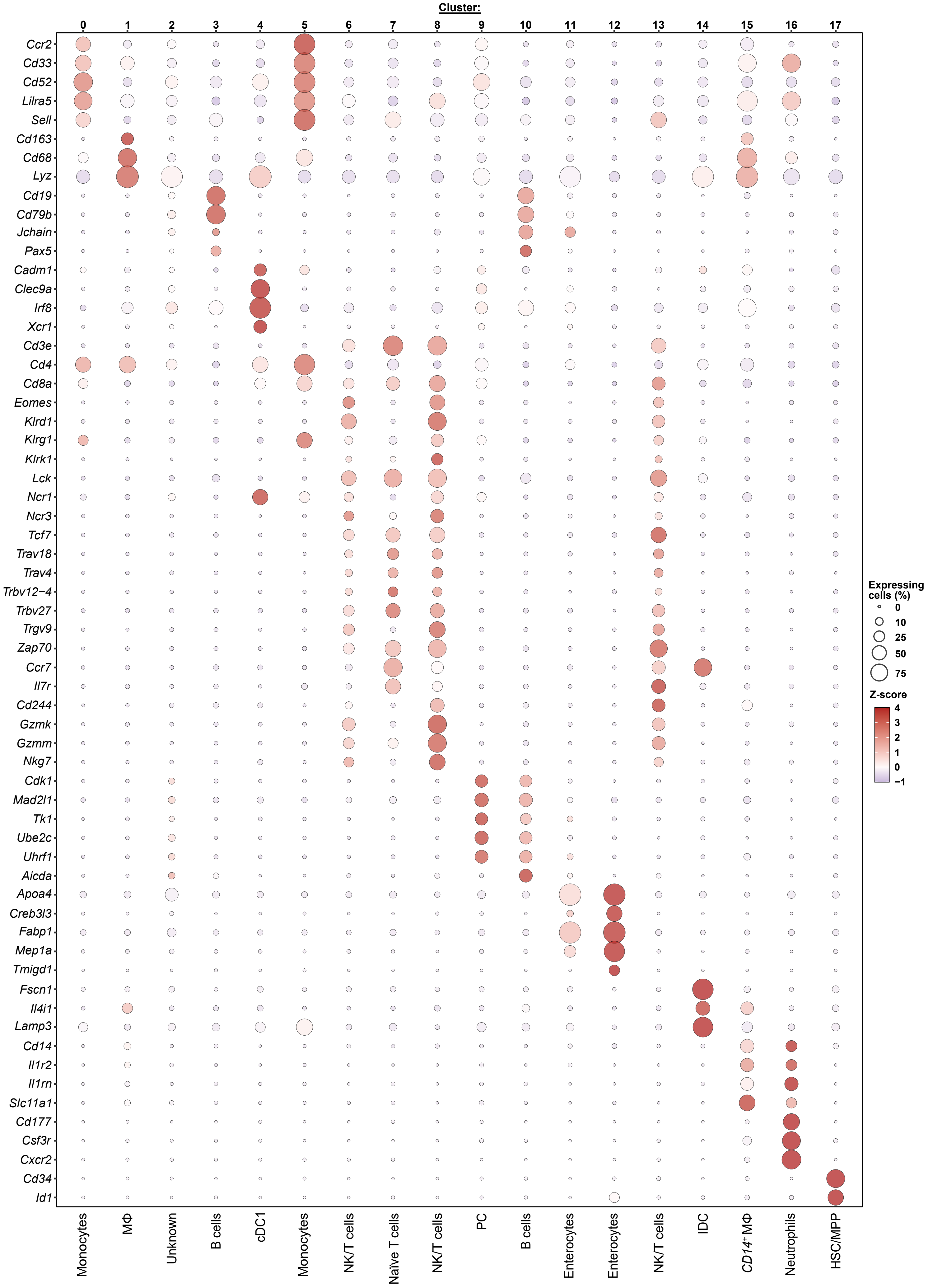

Supplementary Figure 6

Supplementary Fig. 6: Mesenteric cell types defined in the study. Dot plot showing the expression of marker genes of mesenteric cell types in the scRNA-seq dataset. Color scale shows the normalized log_2 expression. Source data are provided as a Source Data file.

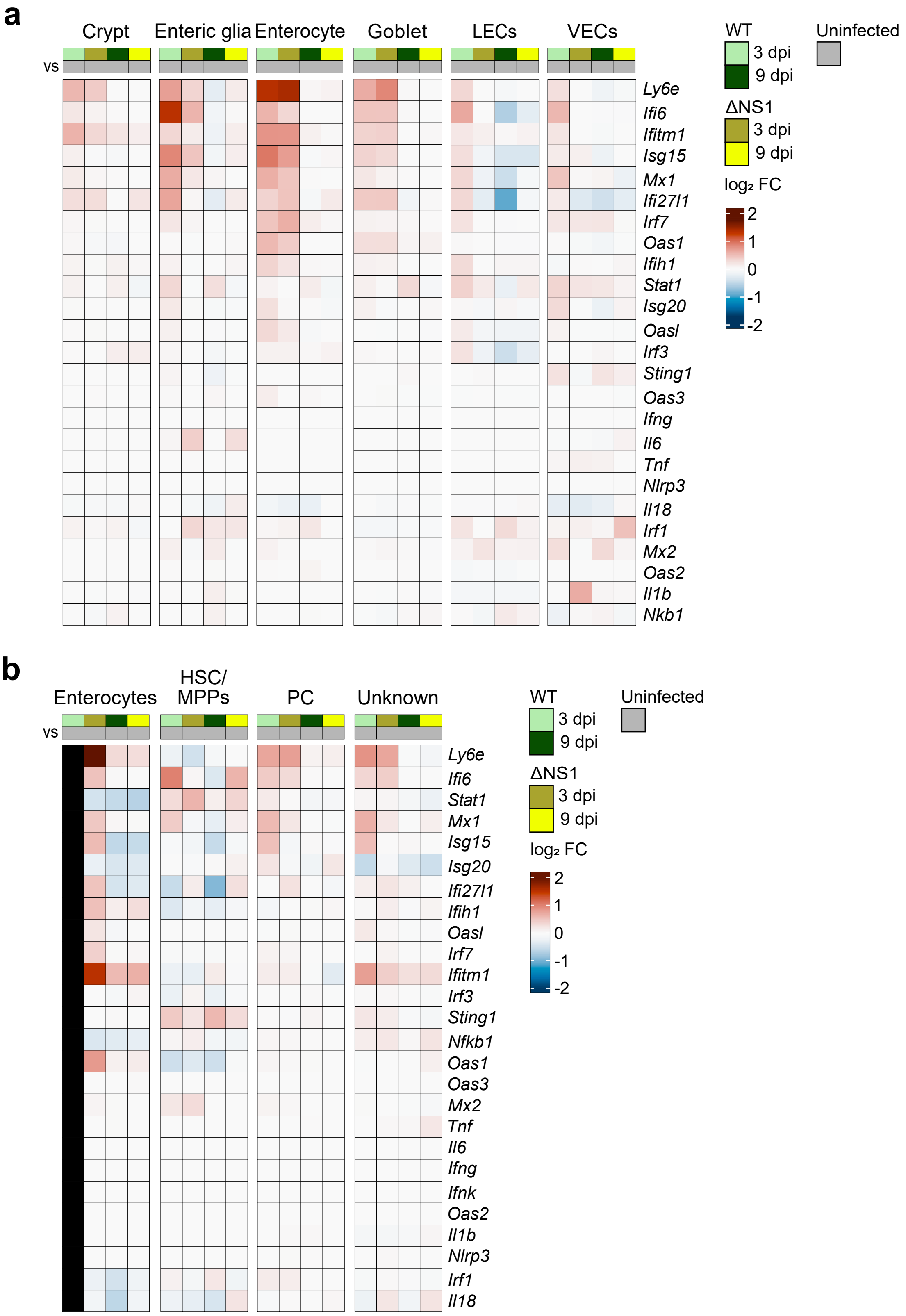

Supplementary Fig. 7: Antiviral gene signatures in intestinal and mesenteric cell types. a,**b**, Gene-level heatmaps showing $\log_2$ fold-changes relative to uninfected controls in interferons and pro-inflammatory cytokines (*Ifng*, *Ifnk*, *Il1b*, *Il6*, *Il18*, and *Tnf*), transcription factors involved in immunity and inflammation (*Irf1*, *Irf3*, *Irf7*, *Nfkb1*, and *Stat1*), ISGs (*Ifi6*, *Ifi27l1*, *Ifitm1*, *Isg15*, *Isg20*, *Ly6e*, *Mx1*, *Mx2*, *Oas1*, *Oas2*, *Oas3*, and *Oasl*) and genes encoding pattern recognition receptors or nucleic acid sensors (*Ifih1*, *Nlrp3*, and *Sting1*) in indicated cell types of the intestine (**a**) and mesentery (**b**). Source data are provided as a Source Data file.

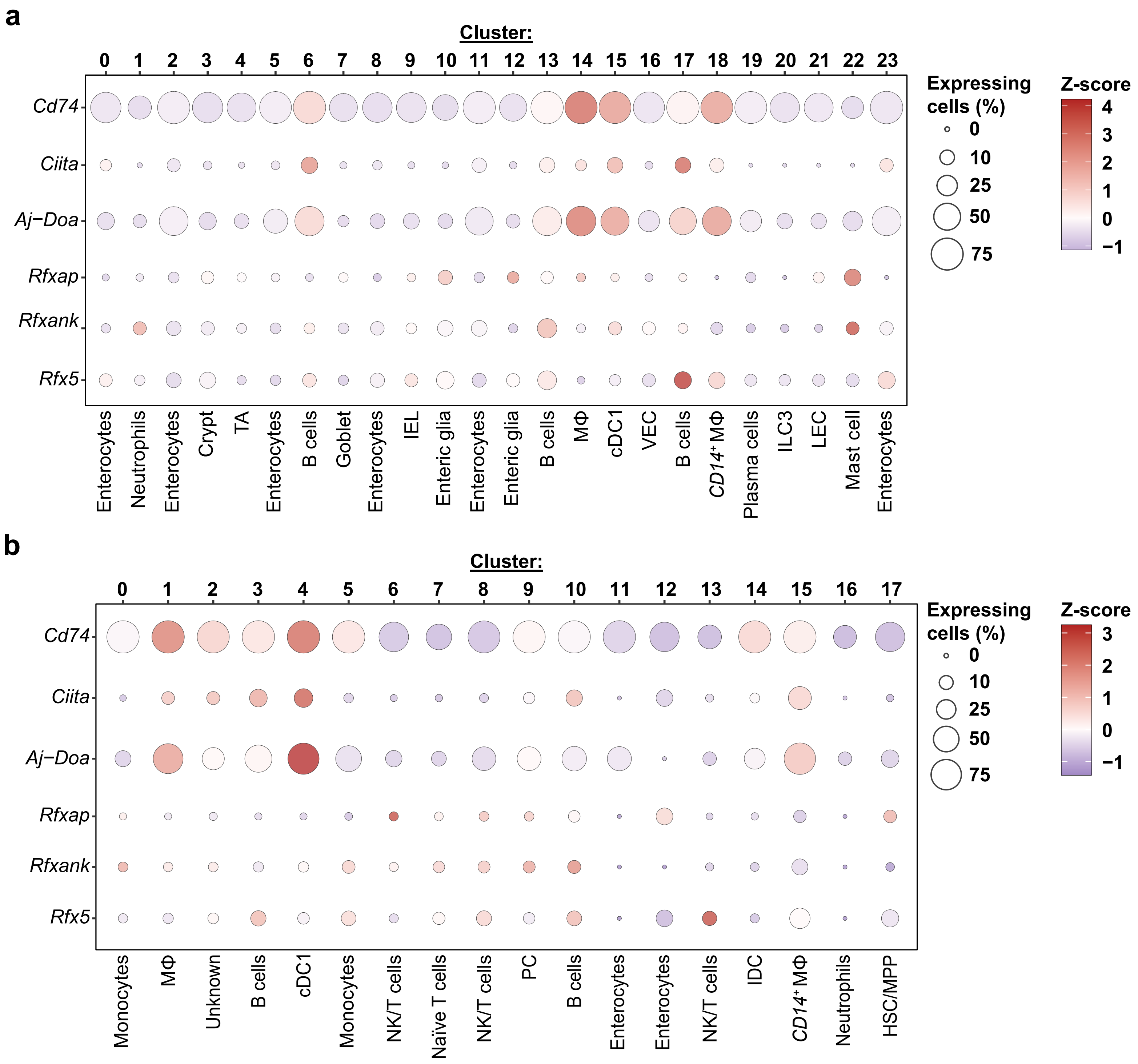

Supplementary Fig. 8: Expression of MHC-II related genes in intestinal and mesenteric cell types. a,**b**, Dot plot showing the gene expression of MHC-II transcription factors (*Rfx5*, *Rfxap*, *Rfxank*, and *Ciita*), the MHC-II-associated invariant chain (*Cd74*) and the alpha chain of the non-classical MHC-II chaperone Aj-DO (*Aj-Doa*) in intestinal (**a**) and mesenteric (**b**) cells. Color scale shows the row-wise scaled mRNA intensity (Z-score) and circle size depends on the percentage of cells expressing a respective gene. Source data are provided as a Source Data file.
