# Supplementary material for: Deciphering bat influenza H18N11 infection dynamics in male Jamaican fruit bats on a single-cell level
Source: Nat Commun. 2024 May 27;15:4500. doi: 10.1038/s41467-024-48934-6 (PMC11130286; doi:10.1038/s41467-024-48934-6)
Supplement: Supplementary file 3 — Description of Additional Supplementary Files [file 41467_2024_48934_MOESM3_ESM.pdf]

Description of Additional Supplementary Files

Supplementary Data 1: Cell type-specifc marker genes. Summary of all marker genes that were used to annotate the intestinal and mesenteric cell clusters of the Jamaican fruit bat.

Supplementary Data 2: Group and sample-wise cell counts for each cell type. Listed are the total cell counts for each cell type and condition, as well as the frequencies calculated for the indicated cell types.

Supplementary Data 3: Regulation of hallmark gene-sets between bat groups. Up- and down-regulated marker genes belonging to the indicated hallmark gene sets are listed.
