# Supplementary material for: Deciphering bat influenza H18N11 infection dynamics in male Jamaican fruit bats on a single-cell level
Source: Nat Commun. 2024 May 27;15:4500. doi: 10.1038/s41467-024-48934-6 (PMC11130286; doi:10.1038/s41467-024-48934-6)
Supplement: Supplementary file 7 — Reporting Summary [file 41467_2024_48934_MOESM7_ESM.pdf]

nature portfolio

Corresponding author(s): Kevin Ciminski
Tony Schountz

Last updated by author(s): May 6, 2024

Reporting Summary

Nature Portfolio wishes to improve the reproducibility of the work that we publish. This form provides structure for consistency and transparency in reporting. For further information on Nature Portfolio policies, see our Editorial Policies and the Editorial Policy Checklist.

Statistics

For all statistical analyses, confirm that the following items are present in the figure legend, table legend, main text, or Methods section.

n/a	Confirmed	
☐	☒	The exact sample size (*n*) for each experimental group/condition, given as a discrete number and unit of measurement
☐	☒	A statement on whether measurements were taken from distinct samples or whether the same sample was measured repeatedly
☐	☒	The statistical test(s) used AND whether they are one- or two-sided<br>*Only common tests should be described solely by name; describe more complex techniques in the Methods section.*
☒	☐	A description of all covariates tested
☐	☒	A description of any assumptions or corrections, such as tests of normality and adjustment for multiple comparisons
☐	☒	A full description of the statistical parameters including central tendency (e.g. means) or other basic estimates (e.g. regression coefficient) AND variation (e.g. standard deviation) or associated estimates of uncertainty (e.g. confidence intervals)
☒	☐	For null hypothesis testing, the test statistic (e.g. *F*, *t*, *r*) with confidence intervals, effect sizes, degrees of freedom and *P* value noted<br>*Give P values as exact values whenever suitable.*
☒	☐	For Bayesian analysis, information on the choice of priors and Markov chain Monte Carlo settings
☒	☐	For hierarchical and complex designs, identification of the appropriate level for tests and full reporting of outcomes
☒	☐	Estimates of effect sizes (e.g. Cohen's *d*, Pearson's *r*), indicating how they were calculated

Our web collection on statistics for biologists contains articles on many of the points above.

Software and code

Policy information about availability of computer code

Data collection	CellSens (v1.18), Leica Application Suite X (LAS-X 3.5.7), ZEISS ZEN softwareCellSens (v1.18), Leica Application Suite X (LAS-X 3.5.7), ZEISS ZEN software, BD FACSDiva Software v.8.0.1
Data analysis	FlowJo v10.8.1. software, R 4.2.1, Seurat R package (v 4.2.0), Prism GraphPad 9, Photoshop 2023, Huygens Professional (v 22.10, SVI), Imaris (v 9.9.1)

For manuscripts utilizing custom algorithms or software that are central to the research but not yet described in published literature, software must be made available to editors and reviewers. We strongly encourage code deposition in a community repository (e.g. GitHub). See the Nature Portfolio guidelines for submitting code & software for further information.

Data

Policy information about availability of data

All manuscripts must include a data availability statement. This statement should provide the following information, where applicable:

- Accession codes, unique identifiers, or web links for publicly available datasets
- A description of any restrictions on data availability
- For clinical datasets or third party data, please ensure that the statement adheres to our policy

The scRNA-seq data generated in this study has been deposited in NCBI’s Gene Expression Omnibus and accessible through GEO Series accession number

GSE243982 (https://www.ncbi.nlm.nih.gov/geo/query/acc.cgi?acc=GSE243982). This paper does not report original code. The data generated in this study are provided in the Supplementary Information/Source Data file.

Research involving human participants, their data, or biological material

Policy information about studies with human participants or human data. See also policy information about sex, gender (identity/presentation), and sexual orientation and race, ethnicity and racism.

Reporting on sex and gender	Human PBMC-derived macrophages from three different adult female study participants were used to determine susceptibility to H18N11.
Reporting on race, ethnicity, or other socially relevant groupings	N/A
Population characteristics	Three healthy female participants with an median age of 28 years were recruited at the University Medical Center Freiburg. Genotypic information was not taken into account.
Recruitment	Healthy donors were recruited at the University Medical Center Freiburg; self-selection bias or other biases can be excluded since several people were included in the recruitment.
Ethics oversight	Written informed consent was obtained from all participants and the study was conducted according to federal guidelines, local ethics committee regulations (Albert-Ludwigs-Universität, Freiburg, Germany). Ethical approval was granted by the Ethics Committee of the University Medical Center Freiburg, reference number 20-1109. Ethical approval 20-1109 of the Ethics Committee of the University Medical Center Freiburg allows phenotyping and functional analysis of lymphocytes from healthy individuals. All participants provided written consent form prior to sample and data collection. All participants provided permission for sample banking and sharing.

Note that full information on the approval of the study protocol must also be provided in the manuscript.

Field-specific reporting

Please select the one below that is the best fit for your research. If you are not sure, read the appropriate sections before making your selection.

☒ Life sciences ☐ Behavioural & social sciences ☐ Ecological, evolutionary & environmental sciences

For a reference copy of the document with all sections, see nature.com/documents/nr-reporting-summary-flat.pdf

Life sciences study design

All studies must disclose on these points even when the disclosure is negative.

Sample size	No sample-size calculations were performed. Sample size was determined to be adequate based on the magnitude and consistency of measurable differences between groups.
Data exclusions	Single Cell-Sequencing data from the mesentery of two animals (x) were excluded from the analysis due to poor quality.
Replication	For all major experiments at least three independent experiments were done and in all cases results could be reproduced. The number of repeats for each experiment is reported in the figure legends.
Randomization	Allocation of bats to groups was random.
Blinding	Investigators were not blinded to the experimental analyses, as this was deemed unnecessary for sample processing, the data collection and analysis.

Reporting for specific materials, systems and methods

We require information from authors about some types of materials, experimental systems and methods used in many studies. Here, indicate whether each material, system or method listed is relevant to your study. If you are not sure if a list item applies to your research, read the appropriate section before selecting a response.

Materials & experimental systems

n/a	Involved in the study
☐	☒ Antibodies
☐	☒ Eukaryotic cell lines
☒	☐ Palaeontology and archaeology
☐	☒ Animals and other organisms
☒	☐ Clinical data
☒	☐ Dual use research of concern
☒	☐ Plants

Methods

n/a	Involved in the study
☒	☐ ChIP-seq
☐	☒ Flow cytometry
☒	☐ MRI-based neuroimaging

Antibodies

Antibodies used

Monoclonal mouse anti-NP; In house (1:500)
Polyclonal rabbit anti-H18; In house (1:500)
AlexaFluor 488 goat anti-Mouse; Jackson ImmunoResearch; #115-546-062 (1:500)
Cy3 goat anti-Rabbit; Jackson ImmunoResearch; #111-165-003 (1:500)
PeCy7 mouse anti-human CD11b; Invitrogen; #25-0118-42 (1:400)
FITC mouse anti-human HLA-DR; BioLegend; #361603 (1:100)
Monoclonal mouse anti-H18; In house (1:500)
BV605 mouse anti-human CD3; BioLegend; #344836 (1:100)
PerCP-Cy5.5 mouse anti-human CD19; BioLegend; #302230 (1:100)
Polyclonal rabbit anti-CD20; Thermo Fisher Scientific; #PA5-1 6701 (1:2.5)
Polyclonal anti-wide spectrum cytokeratin; Abcam; #ab9377 (1:2.5)
Polyclonal rabbit anti- AIF-1/Iba-1; Novus Biologicals; #NBP2-19019 (1:18.5)
Polyclonal rabbit anti-Helicobacter pylori; Dako; #B471 (1:500)
AlexaFluor 488 donkey anti-rabbit; Dianova; #711-545-152 (1:500)
Goat Anti-Rabbit IgG Antibody (H+L); Biotinylated Vector Laboratories; #VEC-BA-1000 (1:200)

Validation

Monoclonal mouse anti-NP was validated on H18N11-infected and mock-infected cells
Polyclonal rabbit anti-H18 was validated on H18N11-infected and mock-infected cells
AlexaFluor 488 goat anti-Mouse was validated by omission of the primary antibody
Cy3 goat anti-Rabbit was validated by omission of the primary antibody
PeCy7 mouse anti-human CD11b was validated with CD11b expressing and non-expressing cells; see also validation statement in the manufacturer's website (www.thermofisher.com)
FITC mouse anti-human HLA-DR was validated with HLA-DR expressing and non-expressing cells; see also validation statement in the manufacturer's website (www.biolegend.com)
Monoclonal mouse anti-H18 was validated on H18N11-infected and mock-infected cells
BV605 mouse anti-human CD3 was validated with CD3 expressing and non-expressing cells; see also validation statement in the manufacturer's website (www.biolegend.com)
PerCP-Cy5.5 mouse anti-human CD19 was validated with CD19 expressing and non-expressing cells; see also validation statement in the manufacturer's website (www.biolegend.com)
Polyclonal rabbit anti-CD20, anti-wide spectrum cytokeratin and anti- AIF-1/Iba-1 antibodies were evaluated by replacing them with an irrelevant primary antibody (Polyclonal rabbit anti-Helicobacter pylori)
AlexaFluor 488 donkey anti-rabbit was validated by omission of the primary antibody
Goat Anti-Rabbit IgG Antibody (H+L) was validated by omission of the primary antibody

Eukaryotic cell lines

Policy information about cell lines and Sex and Gender in Research

Cell line source(s)

HEK293T cells were purchased from the American Type Culture Collection (ATCC); #CRL-3216
MDCKII cells were purchased from ATCC and described previoulsy (https://doi.org/10.1038/s41586-019-0955-3).

Authentication

None of the cell lines used were authenticated.

Mycoplasma contamination

Cell lines are regularly screened for mycoplasma contamination. Cells were not contaminated with mycoplasma.

Commonly misidentified lines
(See ICLAC register)

Name any commonly misidentified cell lines used in the study and provide a rationale for their use.

Animals and other research organisms

Policy information about studies involving animals; ARRIVE guidelines recommended for reporting animal research, and Sex and Gender in Research

Laboratory animals

1-5 years old male Jamaican fruit bats (Artibeus jamaicensis).

Wild animals	This study did not involve wild animals.
Reporting on sex	Although in principle no sex difference was expected in our study, only male animals were used because early stages of pregnancy are not visible in female bats, but could lead to immunosuppression and affect the study results.
Field-collected samples	This study did not involve samples collected in the field.
Ethics oversight	Colorado State University (CSU) Institutional Animal Care and Use Committee, protocol 1574.

Note that full information on the approval of the study protocol must also be provided in the manuscript.

Flow Cytometry

Plots

Confirm that:

☒ The axis labels state the marker and fluorochrome used (e.g. CD4-FITC).

☒ The axis scales are clearly visible. Include numbers along axes only for bottom left plot of group (a 'group' is an analysis of identical markers).

☒ All plots are contour plots with outliers or pseudocolor plots.

☒ A numerical value for number of cells or percentage (with statistics) is provided.

Methodology

Sample preparation	see Online Methods for details<br><br>PBMCs:<br>Human peripheral blood mononuclear cells (PBMCs) were isolated from venous blood of healthy donors by density gradient centrifugation with a Ficoll® Paque Plus (Cytiva) gradient. Freshly isolated PBMCs were infected with WT H18N11 or ΔNS1 in infection medium at an MOI of 5 by spin-infection for 90 min at 1,600 x g and 37°C. Subsequently, the inoculum was removed and replaced by fresh infection medium and cells were cultured at 37°C and 5% CO2. At 24 hpi, cells were harvested and stained and fixed for flow analysis.<br>Monocyte-derived Macrophages:<br>Human PBMCs were resuspended in macrophage growth medium. The next day, cells were washed once and fresh macrophage growth medium was added. Cells were cultured for 7 to 8 days with exchange of medium every two days. Monocyte-derived macrophages were infected with H18N11 in infection medium at the indicated MOI for 1 h at 37°C and 5% CO2. Subsequently, the inoculum was removed and the cells were washed with PBS, followed by a pH shock with PBS pH 2 for 30 seconds and two more washing steps with PBS. Fresh infection medium was added and cells were incubated at 37°C and 5% CO2. At 24 hpi, cells were detached using accutase and mechanical force, stained and fixed for flow analysis.
Instrument	BD LSRFortessa™ Cell Analyzer
Software	BD FACSDiva Software v.8.0.1, FlowJo v10.8.1. software
Cell population abundance	Abundance of infected cells in the different cell types (PBMCs, Macrophages) is shown in the relevant figure (Fig. 6)
Gating strategy	See Fig. 6 for gating strategy. FSC-SSC gating was used to determine cell population and eliminate debris. SSC-H and -A gating was used to exclude duplets. Cell types were identified by staining for prominent markers (CD3, CD19, CD11b). Within these populations the infected cells were stained with an antibody against the viral protein H18. To analyse the viability, Zombie fixable dyes fitting into the panel were used.

☒ Tick this box to confirm that a figure exemplifying the gating strategy is provided in the Supplementary Information.
